# US Farm households: joint decision making and impact of health insurance on labor market outcomes

**DOI:** 10.1186/2191-1991-3-16

**Published:** 2013-05-29

**Authors:** Latika Bharadwaj, Jill Findeis, Sachin Chintawar

**Affiliations:** 1 Research and Statistics Division, Louisiana Workforce Commission, 1001 North 23rd Street, Baton Rouge 70804, USA; 2 Department of Agricultural and Applied Economics, University of Missouri-Columbia, 215 Gentry Hall, Columbia, MO 65211, USA

## Abstract

The paper attempts to answer a very simple question: how does a farm household respond as a unit in the labor market when benefits or health insurance is tied to employer provided jobs. One of the major changes affecting US agriculture has been a decline in the number of farms and an increase in the multiple job-holding, especially among farm women to fulfill various objectives ranging from helping out with farm expenses or securing benefits like health insurance. In addition to this, the new health care law or “The Patient Protection and Affordable Care Act (PPACA”) to be operational by 2014 requires that all individuals be covered by a health plan. Hence, it’s important to understand the relationship between health insurance and labor markets to appropriately identify the impact of health policy reform for farm families.

## Background

The paper attempts to answer a very simple question: how does a farm household respond as a unit in the labor market when benefits or health insurance is tied to employer provided jobs. It’s important to analyze the issue as one of the major changes affecting US agriculture has been a decline in the number of farms and an increase in the multiple job-holding by farm operators, especially farm women to fulfill various objectives ranging from helping out with farm expenses or securing benefits like health insurance [1-4]. Several studies have shown that off-farm employment stabilizes household income and helps to diversify their income risks [5,6]. In addition to this, the new health care law or “The Patient Protection and Affordable Care Act (PPACA)” to be operational by 2014 requires that all individuals not covered by an employer sponsored health plan, medicaid, medicare or other public insurance programs, secure an approved private insurance policy or pay a penalty [7]. Hence, it’s important to understand the relationship between health insurance and labor markets to appropriately identify the impact of health policy reform for farm families. In this study, we present findings related to joint decision making among farm households and dynamic adjustment among farm women and spouse partner related to off-farm labor participation with or without benefits depending on the needs of the farm family. In most cases, if the spouse/partner is working full-time with health insurance, then the farm women is more likely to work part-time or full-time without insurance perhaps to socialize with other people or be independent, depending on the situation of the farm household. However, in households where the spouse works on the farm, farm women are most likely to be working at full-time job that provides health insurance, thereby saving money from buying benefits for the huge farm operation.

Off-farm work among farm families has become so important that one can question the conventional view that work time is first allocated to farming and then, if there is time left, to off-farm work [8]. In the conventional view, it is assumed that the initial allocation of labor on the farm has a higher marginal productivity and exceeds the market wage rate. It is only when the on-farm value of the marginal product of labor becomes equal to the off-farm market wage rate, that the farmer assigns labor to off-farm work [9,10]. But several studies have pointed out [8,11-13] that due to higher marginal returns in off-farm work, some farm households may first allocate labor to non-farm work. In fact, this view becomes even more relevant when we look at the cost of health insurance, and increasing participation of farm households especially farm women in off-farm labor markets to provide the farm family financial protection, such as health insurance that is generally not economical for the farm business to purchase for family members [14,15]. However, there are very few studies on farm households, and the effect of employer-provided health insurance on labor participation, especially among households where farm women or both spouses might be receiving health insurance. In light of the above stated background, this paper tries to look at how farm families respond to off-farm jobs with benefits like health insurance.

Now, health insurance is unique in the US because of the way it is offered in the labor market. Due to tax savings associated with the exemption of health benefits from federal and state taxation, private employers are the major providers of health insurance in the U.S. [16]. But this trend is changing, and studies have found a decline, both in offer rates (the percentage of workers offered health benefits) and the coverage rates for employment based health benefits [17,18]. There are several issues associated with the provision of health insurance and the resulting distortions caused in the labor market. According to the Internal Revenue Services, fringe benefits offered by a firm should not discriminate against the firm’s low-wage employees. As a result, health insurance packages cannot be tailored to the needs of individual employees^a.^ Another issue is that fringe benefits have to be offered in a nondiscriminatory manner. For example, high-wage firms, which tie pension benefits to the earnings of the worker, avoid hiring low-wage workers, as they have to offer all full-time workers the same health benefits [19]. As a result, health insurance is mostly offered to ***full-time high-wage*** workers rather than ***part-time low-wage*** workers [17]. However, these provisions might be changing with the new health care act. Employers with fewer than 50 employees are not required to offer health insurance to their workers. But if they do, then they may qualify for a tax credit to provide health insurance. Conversely, medium and large businesses that employ more than 50 workers will be required to provide affordable health insurance coverage to their employees. Failure to do so will result in an assessed penalty of $2,000 to $3,000 per employee, excluding the first 30 employees [7]. However, employer responses to new health care act are beyond the scope of this paper, since the survey does not cover any employer characteristics.

The above outlined changes have huge implications for farm families especially the likelihood of receiving benefits when one is low wage or part-time, but eligible for subsidies through health exchanges. Most of the times it has been found that the farm woman (or man) works off the farm for benefits and farm man (woman) works on the farm. Farm women might even work part-time at the non-farm job depending on different circumstances where she might be either helping out the farm or looking after small children or due to her skill set, is classified as a low wage worker. If this is the situation, then does she receive any benefits or not? Much of the literature until now has focused on issues related to impacts of health insurance on retirement and job mobility (for example, see [20-23]). A few studies have also examined issues related to labor supply of lower-income single mothers and labor supply of married couples [23]. Studies related to married women’s labor supply have concluded that the husband’s health insurance has a strong negative effect on the wife’s work hours, especially among families with children, and that it is mostly women without spousal health insurance who will work full-time in the labor market rather than women with spousal health insurance [24]^b^; [25]^c^. But very few studies have focused on farm families and joint decision making among farm couples related to off-farm labor participation especially in relation to farm women seeking off-farm jobs with benefits like health insurance. Hence, in this paper, we try to analyze, how the link between employer provided health insurance and joint decision making within farm households, affects the labor market choices such as to work full time or part time. In the light of ‘new health care act’, understanding joint decision making in farm households in the context of health insurance will help us identify how much health insurance drives labor market decisions when access to health insurance depends on the choice made.

As result, this study first analyzes household decision making process in regards to off-farm participation (and if it involves joint decision among farm couples) and then depending on that decision looks at individual or joint equations for examining factors determining benefit receipt of the farm household. If joint decision making is ignored and independent labor participation decisions are considered for farm husband and farm wife, then there is possible danger of losing interdependence with spouse decision with respect to off-farm labor and fringe benefits. Bivariate Probit models are estimated for off-farm participation equations followed by multinomial logit models to determine factors affecting benefit receipt from off-farm work.

The paper uses data from a survey titled “The Women on Farms Survey (2001)” conducted by Pennsylvania State University in collaboration with researchers at the Economic Research Service (ERS, USDA) and the National Agricultural Statistics Service (NASS, USDA). The next three sections lay out the theoretical framework, data and the estimation strategy for the paper. Finally the last section concludes with a discussion of the results.

## Methods

The agricultural household model analyzes the three decisions related to production, consumption and work decisions in one framework. It is an extension of the simple household goods-leisure decision model in which household members maximize total utility of the household, under the constraints of total household income and total endowment time. To provide for goods and services, some members of the household typically have to work (unless there is adequate income from non-earned sources such as rent or transfers of family wealth). Trade-offs are made between the consumption of goods and services and the amount of leisure time enjoyed by the household members.

Following Huffman [9] consider a farm household consisting of two members’ *m* and *f* who can choose to work on household’s farm or to work off-farm. The utility function is maximized subject to the following constraints stated below.

(1.1) $$\mathrm{Max}\;U=U\left(C,L^{m},L^{f}\right)$$

Subject to

(1.2) $$\begin{array}{l} Q=\left(F^{m},F^{f},X;A,\Pi ,\Omega \right)\\ \;\left(\mathrm{Production}\;\mathrm{function}\;\mathrm{constraint}\right) \end{array}$$

(1.3) $$\begin{array}{l} P_{q}Q-P_{x}X+W^{m}M^{m}+W^{f}M^{f}+I\leq P_{c}C\\ \;\left(\mathrm{Budget}\;\mathrm{constraint}\right) \end{array}$$

(1.4) $$\begin{array}{cc} T^{i}=L^{i}+F^{i}+M^{i} & \left(\mathrm{Time}\;\mathrm{constraint}\right) \end{array}$$

(1.5) $$\begin{array}{cc} F^{i}\geq 0,\;M^{i}\geq 0 & \left(\mathrm{non-negativity}\;\mathrm{constraint}\right) \end{array}$$

Where *i* = *m*, *f*.

*C* is vector of consumption goods, *Q* is farm output produced, *X* = variable inputs used in farm production, *A* is fixed quantity of land, ∏ = vector of household characteristics, Ω is farm specific exogenous characteristics, *P*_*c*_ = price of consumption goods, *P*_*q*_ = price of farm outputs, *P*_*x*_ = price of variable inputs, *W*^*i*^ = market wage for individual *i*, *L*^*i*^ = time allocated to leisure by individual *I, F*^*i*^ = time allocated to farm work by individual *I, M*^*i*^ = time allocated to market work by individual *i*, *T*^*i*^ = total time available to individual *i.* The utility function is assumed to be twice differentiable, i.e., *U*_*i*_ > 0 and *U*_*ii*_ < 0, where i represents the arguments of the utility function.

### Data

**Figure 1:**
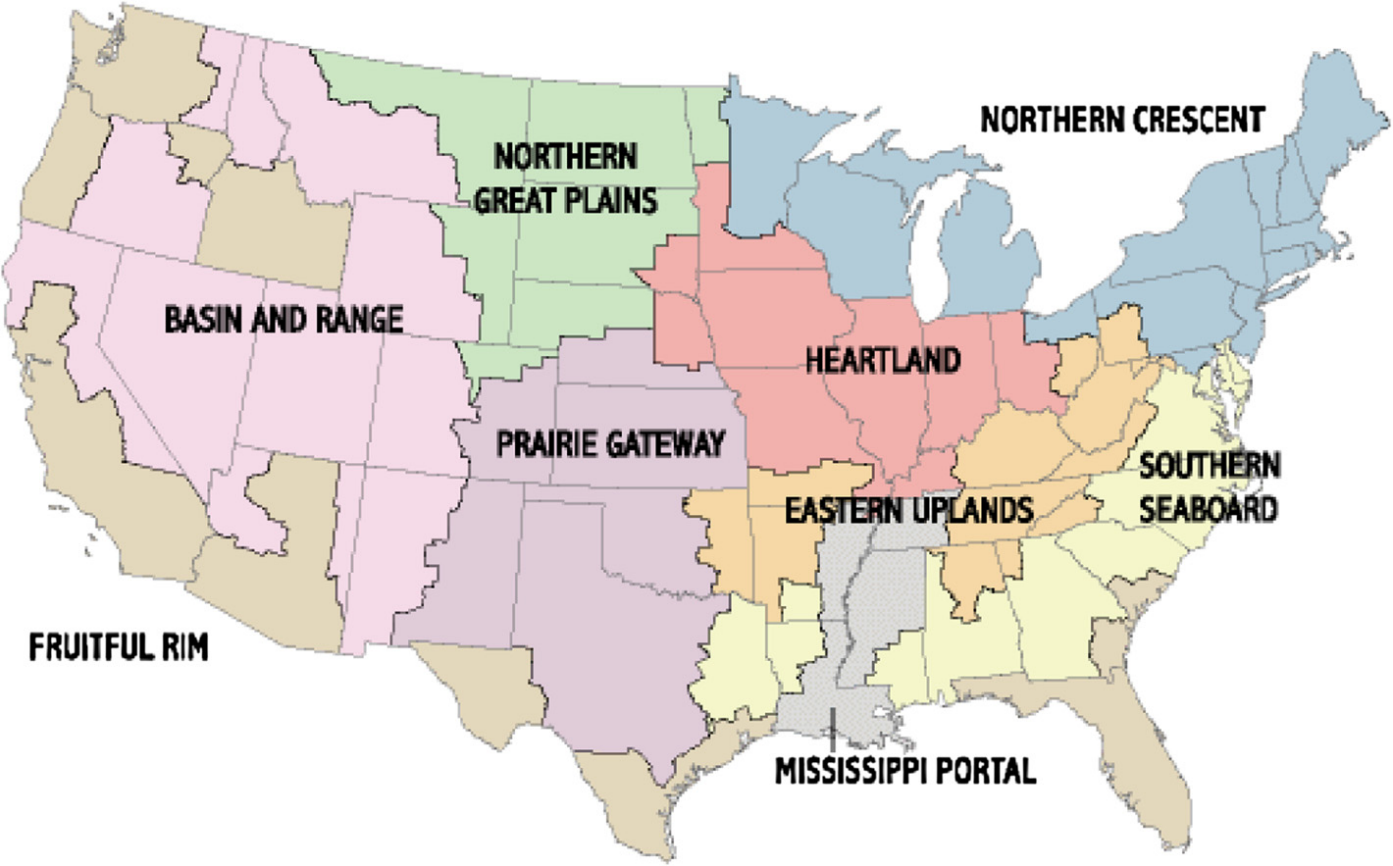
Farm resource regions.

The data used for this study have been taken from a survey titled “The Women on Farms Survey (2001)” collected by Penn State in collaboration with the Economic Research Service (ERS) and the National Agricultural Statistics Service (NASS) at the U.S. Department of Agriculture. Rosenfeld collected the last major survey on farm women in 1980 (see [26]). This survey was carried out in April 2001 by telephone and a national sample of 7,500 farms was selected by NASS. A sum of 2,661 farm women responded to the survey corresponding to a response rate of 35 percent. A small subset of farm men was also included in the survey, so that joint-decision making can be understood in a better way. The survey focused on questions related to farm and off-farm work, motivation to work off-farm and benefits received from off-farm work. Other questions asked were related to operation of the farm and demographic characteristics of the farm household. Data from Regional Economic Information System of the Bureau of Economic Analysis (REIS/BEA) and the 2000 census for the U.S. has been appended to the main dataset, so that information is available on variables related to off-farm labor market like commuting zone population growth rate and commuting zone unemployment rate. The survey data also includes information on county of residence, which was used as a basis to match the data with the nine productions regions identified by Economic research service, U.S.D.A (Figure 1).

### Estimation strategy: participation in off-farm work

First factors affecting the participation in an off-farm work is examined. The Women on Farms Survey (2001) asked farm women and their spouses/partners if they worked at an off-farm job during 2000^d^. Hence, factors affecting participation in off-farm work are examined for both the farm woman and her spouse/partner^e^. When there is potential jointness in decisions, bivariate probit models can be estimated to analyze off-farm work participation decisions, with presence of jointness depending on statistical significance of rho (*ρ*) or the correlation of the error terms, i.e. if there is no correlation then univariate probits can be used and if the stochastic errors terms for factors affecting decision to work off-farm by farm women and their spouse are associated with each other, then appropriate statistical model will be bivariate probit model.

### Discussion of variables: participation in Off-farm work

Descriptive statistics of the variables used in the estimation of the off-farm labor participation equations are included in Table 1 for the farm woman and her spouse/partner, respectively. The tables are for the full sample. The tables show differences in means by off-farm work status, i.e., if employed off the farm (at all) in the past year (2000) or not. Age may affect off-farm labor participation decisions and the hours of work supplied of the farm man and woman differently, i.e., the influence of age may not be consistent across gender. Most studies of farm women in Europe conclude that the probability of wage work participation decreases at older ages and participation in off-farm work is higher for younger farm women than older ones [27-29]. The means for the 2001 Penn State survey show that the average age of farm women working at off-farms jobs is 48 for farm women (Table 1), and 50 for the spouse/partner (Table 1). The average ages for those not working off-farm are substantially higher, in part because of the influence of retirement. Both the age and age-squared variables will be incorporated in the specifications for this research to capture the expected life-cycle curvilinear effect in labor participation for both the farm women and her spouse/partner.

**Table 1:** Descriptive statistics for labor participation models for farm woman and farm husband/spouse partner

	**Means**			
**Variable**	**Woman with off-farm job**	**Woman without off-farm job**	**Spouse with off-farm job**	**Spouse with no off-farm job**
**Age (years)**				
Age	48.14	55.62	50.63	58.08
Age squared	2400.21	3246.91	2666.24	3526.47
**Presence of**:				
Children under 6 years of age (1 = yes)	0.11	0.10	0.13	0.08
Children age 6 to 11 years (1 = yes)	0.16	0.16	0.17	0.14
Children age 12 to 18 years (1 = yes)	0.31	0.18	0.30	0.20
Children over 18 years and away (1 = yes)	0.31	0.19	0.30	0.21
**Educational attainment**				
High school graduate	0.35	0.43	0.42	0.42
Vocational/technical school/some college	0.30	0.29	0.29	0.25
College graduate	0.20	0.13	0.11	0.12
Post graduate				
*Reference category:* Less than high school	0.12	0.05	0.11	0.06
**Labor market characteristics**				
Commuting zone unemployment rate (2000)	4.0	4.0	4.0	4.0
Commuting zone population growth rate (1990–2000)	10.8	11.1	11.6	10.2
**Farm inherited or purchased:**				
Through her family	0.14	0.14	0.13	0.16
Through his family	0.28	0.30	0.26	0.32
**Growing up years for farm woman**				
In country, but not on farm	0.13	0.13	1.89	1.94
In small town	0.25	0.22	1.83	1.89
Suburban or urban area				
*Reference category:* Growing up on a farm or ranch	0.52	0.55	1.88	1.93
**Farm characteristics**				
Land owned (acres)	266.16	585.44	155.05	680.79
Debt-asset ratio	0.35	0.28	0.35	0.28
**ERS farm production regions**				
Northern Crescent	0.16	0.15	0.17	0.15
Eastern Uplands and Mississippi Portal	0.20	0.19	0.23	0.16
Southern Seaboard	0.08	0.12	0.10	0.10
Fruitful Rim	0.09	0.11	0.10	0.10
Basin and Range	0.04	0.06	0.05	0.05
*Reference category:* Heartland, Northern Great Plains, Prairie Gateway				

Information is available on the level of school attended for both the farm man and farm woman from the 2001 Penn State ‘Women on Farms Survey’. Table 1 shows, that as compared to the less than high school category, most farm women and spouses/partners graduated only from high school (35% for farm women and 42% for farm men with off-farm jobs; 43% for farm women and 42% farm men without off-farm jobs). The sample also contains (4-year) college graduates (20% for farm women and 11% for farm men with off-farm jobs; 13% for farm women and 12% for farm men with no off-farm jobs) and post graduates (12% of farm women and 11% of farm men with off-farm jobs; 5% of farm women and 6% of farm men with no off-farm jobs).

Most studies have found that the presence of very young children reduces the likelihood of off-farm work among both farm men and women. Older children could have an ambiguous effect on the off-farm work participation decision of either parent, depending on whether the farm labor of a parent and that of an older child are substitutes or complements. In the estimated models, children of different age groups (less than 6 years of age; 6 to 11 years; 12 to 18 years; children above 18 living at home or children living away from home) have been included for both the farm woman and her spouse/partner.

Among labor market characteristics, the commuting zone unemployment rate and commuting zone population growth rate have been included in the participation equations. Previous studies that have used variables like population density [30,31] and local unemployment rates [32-34], have reported significant effects on participation in local labor markets. Table 1 show rates of 4 percent for the commuting zone unemployment rate (for year 2000) and approximately 11 percent for the commuting zone population growth rate (over the 10-year period from 1990 to 2000).

Acquiring (inheritance or purchase) of the farm through the farm woman’s (spouse’s/partner’s) family may affect participation in a non-farm job. There may be an emotional attachment to the farm, and the farm family may seek to maintain the farm in the family for future generations. Dummy variables are used to indicate the source of purchase or inheritance of the land (i.e., through the woman’s family, husband’s/partner’s family, or others (reference category).

The financial position of the farm family and the characteristics of the farm operation are likely to influence off-farm work decisions. Generally, families with large farm asset values are less likely to work off the farm. This could be an indicator of wealth effect on off-farm labor participation. Hence, the debt-asset ratio and land owned (acres) are used in the labor participation models for both the farm woman and her spouse/partner.

The control variables that reflect the predominant farming systems (as well as other relevant regional characteristics) include dummy variables for the USDA Farm Resource Regions and size of place where the farm woman or spouse/partner grew up. The designation of the regions is based on the nine Farm Resource Regions. Dummy variables are used to indicate if the individual grew up in the country, but not on a farm; in a small town; in a suburban area or urban area, with growing up on a farm or ranch as the reference category.

The method of maximum likelihood is used for estimating the coefficients of the estimators. The principle of maximum likelihood provides a unified approach to estimating parameters of the distribution of the sample data. Additionally it provides desirable asymptotic properties including normality and efficiency (See [35] on a detailed discussion of using maximum likelihood estimation). The independent variables in the model include, individual characteristics like age, age squared, level of schooling (reflecting human capital and experience), household characteristics like presence of children of different age groups, farm characteristics like value of assets or location of the farm in various regions differentiated by U.S.D.A, local labor market conditions like commuting zone unemployment growth rate and population growth rate.

### Estimation strategy: benefit receipt

Based on ***Jointness*** or ***No Jointeness*** in household decision making multinomial logit models will be formulated to examine factors determining benefit receipt of the farm household. If there is no jointness in decision making among the farm couple, then 2 separate equations (one for farm women and other for spouse) using multinomial logit are estimated for determining factors affecting benefit receipt with the following work choices: no work, work in a part-time job with benefits, work in a part-time job without benefits, work in a full-time job with benefits, and work in a full-time job without benefits.

Since there might be jointness in decisions related to off-farm work participation between farm women and her spouse, factors affecting benefit receipt for a household are estimated using both farm women’s and spouses off- farm work hours differentiated by part time and full time work in conjunction with health insurance using a multinomial logit model. Hence, the categories in the multinomial logit are defined by farm woman’s and spouse/partner’s hours of work (categories) and health insurance status. The categories consist of non-participation (no off-farm work), part-time and full-time^f^ off-farm work which are combined with their respective health insurance statuses.

### Discussion of variables: benefit receipt

Table 2 provides break-downs of the numbers of households involved in off- farm work with and without health insurance as well as those not employed in off-farm labor market. There are a total of 1674 households in the working-age category and 62% of the households receive health insurance associated with the off-farm employment of either the farm woman or the spouse/partner or both. About 17% of the households have both the farm woman and spouse/partner working off-farm or at least one person working off-farm, but without any health insurance from this work and approximately 22% of the household’s do not participate in the labor market at all. Since the new health care act becomes operational in 2014, we wanted to explore the impact of this law on households not working in labor market or working in the labor market, but without receiving any employer based benefits. This would also be the base category in multinomial logit estimation where there might be potential jointness in off-farm labor participation. According to the new health care law, low income individuals and families above 100% and up to 400% of the federal poverty level will receive federal subsidies on a sliding scale if they choose to purchase insurance via an exchange (those from 133% to 150% of the poverty level would be subsidized such that their premium cost would be 3% to 4% of income). The Women on Farms Survey (2001) has information about demographic characteristics and financial characteristics of farm households. Hence, we analyze this impact on the farm families by looking at the distribution of farm household size and total income^g^ differentiated by the status of off-farm work and employer benefits. Descriptive statistics of the variables used in the estimation of the multinomial logit equations are included in Table 3.

**Table 2:** Employer-provided benefit receipt by U.S. farm women and spouse/partners for working-age population (18–64), 2001 survey

**Work status with benefits**	**Frequency**	**Percent**
	**(N)**	**(%)**
Farm woman works with HL and spouse/partner works with HL^a^	265	16%
Farm woman works with no HL and spouse/partner works with HL	217	13%
Farm woman works with HL and spouse/partner works with no HL	148	9%
Farm woman no off-farm work and spouse/partner works with HL	181	11%
Farm woman works with HL and spouse/partner no off-farm work	227	14%
**Households receiving any health insurance (HL) associated with off-farm work**	**1038**	**62%**
Farm woman works with no HL and spouse/partner works with no HL	81	5%
Farm woman no off-farm work and spouse/partner works with no HL	60	4%
Farm woman works with no HL and spouse/partner no off-farm work	135	8%
Farm woman no off-farm work and spouse/partner no off-farm work	360	22%
N	1674	100%

HL^a^ = Health Insurance.

**Table 3:** **Descriptive statistics for variables for work status and health insurance categories, 2001 survey**^**1**^

**Variable**	**No off-farm work (Y = 0)**	**Farm woman works part-time with no health insurance & spouse works full-time with health insurance (Y = 1)**	**Farm woman works full-time with health insurance & spouse works full-time with health insurance (Y = 2)**	**Farm woman works full-time with health insurance & spouse works full-time with no health insurance (Y = 3)**
Age (years)	58.730	46.727	45.887	47.000
	(0.477)	(0.924)	(0.595)	(0.906)
**Presence of**:	0.135	0.295	0.212	0.247
Children under 6 years or age 6 to 11 (1 = yes)	(0.014)	(0.049)	(0.029)	(0.046)
Children age 12 to 18 (1 = yes)	0.137	0.420	0.305	0.360
	(0.014)	(0.053)	(0.032)	(0.051)
Children over 18 and away (1 = yes)	0.039	0.080	0.094	0.101
	(0.008)	(0.029)	(0.020)	(0.032)
**Farm woman educational attainment**	0.383	0.295	0.256	0.258
High school graduate	(0.020)	(0.049)	(0.031)	(0.047)
Vocational/technical school/some college	0.416	0.466	0.453	0.438
	(0.020)	(0.053)	(0.035)	(0.053)
College graduate	0.092	0.182	0.197	0.247
	(0.012)	(0.041)	(0.028)	(0.046)
Post graduate	0.045	0.125	0.143	0.146
*Reference Category: Less than high school*	(0.008)	(0.035)	(0.025)	(0.038)
**Farm man educational attainment**	0.415	0.386	0.394	0.449
High school graduate	(0.020)	(0.052)	(0.034)	(0.053)
Vocational/technical school/some college	0.217	0.295	0.320	0.292
	(0.017)	(0.049)	(0.033)	(0.048)
College graduate	0.114	0.182	0.143	0.124
	(0.013)	(0.041)	(0.025)	(0.035)
Post graduate	0.063	0.057	0.089	0.090
*Reference category: Less than high school*	(0.010)	(0.025)	(0.020)	(0.030)
**Farm characteristics**	623.294	133.477	171.463	154.573
Land owned (acres)	(77.142)	(27.170)	(33.628)	(18.283)
**Labor market characteristics**	0.110	0.108	0.121	0.115
Commuting zone population growth rate (1990–2000)	(0.004)	(0.010)	(0.008)	(0.011)
Commuting zone unemployment rate (2000)	0.041	0.039	0.039	0.038
	(0.001)	(0.002)	(0.001)	(0.002)
**ERS farm production regions**	0.143	0.205	0.138	0.124
Northern Crescent	(0.014)	(0.043)	(0.024)	(0.035)
Eastern Uplands and Mississippi Portal	0.175	0.182	0.251	0.213
	(0.015)	(0.041)	(0.031)	(0.044)
Southern Seaboard	0.109	0.068	0.128	0.090
	(0.013)	(0.027)	(0.024)	(0.030)
Fruitful Rim	0.109	0.114	0.064	0.079
	(0.013)	(0.034)	(0.017)	(0.029)
Basin and Range *Reference category: Heartland, Northern Great Plains, Prairie Gateway*	0.050	0.034	0.025	0.022
	(0.009)	(0.019)	(0.011)	(0.016)
Total Income	5.090	5.862	6.288	5.746
	(0.116)	(0.202)	(0.111)	(0.195)
Household size	2.703	3.409	3.113	3.056
	(0.059)	(0.133)	(0.127)	(0.106)
Housel size*Total income (Interaction variable0)	14.673	20.362	19.199	17.841
	(0.574)	(1.313)	(0.673)	(1.013)
**Variable**	**Farm woman works full-time with no health insurance & spouse works full-time with health insurance (Y = 4)**	**Farm woman does not work & spouse works full-time with health insurance (Y = 5)**	**Farm woman works part-time with no health insurance & spouse does not work (Y = 6)**	**Farm woman works full-time with health insurance & spouse does not work (Y = 7)**
Age (years)	46.714	49.050	52.839	49.189
	(0.710)	(0.824)	(1.167)	(0.617)
**Presence of**:	0.245	0.242	0.194	0.189
Children under 6 years or age 6 to 11 (1 = yes)	(0.044)	(0.034)	(0.041)	(0.030)
Children age 12 to 18 (1 = yes)	0.398	0.255	0.269	0.309
	(0.050)	(0.034)	(0.046)	(0.035)
Children over 18 and away (1 = yes)	0.143	0.062	0.065	0.069
	(0.036)	(0.019)	(0.026)	(0.019)
**Farm woman educational attainment**	0.357	0.298	0.301	0.297
High school graduate	(0.049)	(0.036)	(0.048)	(0.035)
Vocational/technical school/some college	0.459	0.472	0.484	0.446
	(0.051)	(0.039)	(0.052)	(0.038)
College graduate	0.153	0.174	0.172	0.171
	(0.037)	(0.030)	(0.039)	(0.029)
Post graduate	0.102	0.037	0.032	0.154
*Reference category: Less than high school*	(0.031)	(0.015)	(0.018)	(0.027)
**Farm man educational attainment**	0.490	0.379	0.398	0.480
High school graduate	(0.051)	(0.038)	(0.051)	(0.038)
Vocational/technical school/some college	0.286	0.255	0.312	0.229
	(0.046)	(0.034)	(0.048)	(0.032)
College graduate	0.143	0.137	0.129	0.149
	(0.036)	(0.027)	(0.035)	(0.027)
Post graduate	0.061	0.155	0.022	0.069
*Reference Category: Less than high school*	(0.024)	(0.029)	(0.015)	(0.019)
**Farm characteristics**	155.306	189.255	737.204	498.480
Land owned (acres)	(35.825)	(45.367)	(214.578)	(99.812)
**Labor market characteristics**	0.096	0.113	0.098	0.099
Commuting zone population growth rate (1990–2000)	(0.010)	(0.009)	(0.009)	(0.009)
Commuting zone unemployment rate (2000)	0.038	0.042	0.038	0.038
	(0.002)	(0.001)	(0.002)	(0.001)
**ERS farm production regions**	0.184	0.174	0.172	0.149
Northern Crescent	(0.039)	(0.030)	(0.039)	(0.027)
Eastern Uplands and Mississippi Portal	0.245	0.248	0.140	0.149
	(0.044)	(0.034)	(0.036)	(0.027)
Southern Seaboard	0.041	0.143	0.043	0.091
	(0.020)	(0.028)	(0.021)	(0.022)
Fruitful Rim	0.071	0.087	0.097	0.057
	(0.026)	(0.022)	(0.031)	(0.018)
Basin and Range *Reference category: Heartland, Northern Great Plains, Prairie Gateway*	0.041	0.056	0.065	0.034
	(0.020)	(0.018)	(0.026)	(0.014)
Total Income	6.088	5.939	4.946	5.864
	(0.193)	(0.174)	(0.288)	(0.159)
Household size	3.265	3.143	2.871	2.943
	(0.128)	(0.121)	(0.126)	(0.083)
Housel size*Total income (Interaction variable0)	21.491	17.602	15.500	17.552
	(1.272)	(0.887)	(1.550)	(0.768)

^1^Standard error in parentheses.

The method of maximum likelihood is used for estimating the coefficients of the estimators. Among the independent variable, individual characteristics such as age have been included in the estimation to test its influence on off-farm work participation with employer-provided health insurance for farm households. Studies have found that among both men and women, offer rates increased with age [17]. However, age of the farm man is not included due to problems of multicollinearity with the farm woman’s age [36]. Studies have also shown that education and work experience are significant predictors of human capital and that receipt of employer-provided benefits tends to rise with skill [25,37,38].

According to a studies by Faber and Levy, [39]) and Fronstin [17], the major cause of a difference in insured and uninsured rates between families was the less than high school education for wage earners in uninsured families to the post-graduate education observed among wage earners from insured families. For this study, ‘The Women on Farm Survey’ provides information related to levels of schooling. Specifically, information is available on five levels of schooling for both the farm woman and her spouse/partner: 1) whether they attended high school, but did not graduate, 2) graduated from high school, 3) graduated from vocational or technical school, 4) graduated with a 4-year college, or 5) attained a post graduate degree. Hence, education is included to estimate the impact of schooling on off-farm labor participation with or without health insurance for the farm woman and spouse/partner.

Farber and Levy [39], in their study related to insurance and health care, conclude that families with children are more likely to be insured, and children among two-parent working families are more likely to have health insurance than children in single-parent working families. Hence, family characteristics are represented by including presence of children of different age-groups (less than 6 years, 6 to 11 years, 12 to 18 years, and children above 18 living at home or away).

Financial resources reflected by farm characteristics can be a key determinant for seeking employment with benefits. A study by French and Jones [21] hypothesizes that individuals with high levels of assets are less sensitive to the impacts of health insurance on retirement, as they can afford to self-insure. Assets such as land owned in acres may affect decisions to work in the off-farm labor market with health insurance for both the farm woman and spouse/partner. The purchase or inheritance of land variables was not found significant in joint labor participation models; hence it was left out from these estimations.

A strong economy indicates strong labor market conditions that lead to increases in labor demand, whereas high unemployment rates may cause firms to reduce benefit packages with high fixed costs [38]. Several studies use geographic regions to explain variations in off-farm jobs with health insurance. For example, Meyer *et al.*, [38] concludes that workers in the Midwest have a higher probability of receiving health insurance but face lower wages in comparison to skilled workers in other regions. The authors show that local labor market conditions do not have much of an impact on wages, but areas with higher average earnings and rising employment have a higher likelihood of receiving employer-provided health insurance. Hence, non-pecuniary benefits more than wages are used to attract or retain workers. In the estimated models commuting zone unemployment rate and commuting zone population growth rates have been included as indicators of labor market function^h^. In addition, the nine resource regions delineated by the USDA are incorporated.

## Results and discussion

Participation in off-farm work, when both farm woman and husband are present, can be a joint decision. Since, there was a correlation in the error terms; bivariate probit models were used for estimating the participation equation. The marginal effects and relevant t-statistics for the participation are presented in Table 4. The statistical package used in the empirical analysis was LIMDEP 8.0.

**Table 4:** **Bivariate probit results for participation in off-farm work, 2001 survey**^**1**^

**Variable**	**Marginal effects farm women**	**Marginal effects spouse partner**
Age (years)	0.055	0.028
	(3.52)***	(1.89)*
Age squared	−0.001	0.0004
	(−4.72)***	(−3.20)***
**Presence of**:	−0.135	−0.035
Children under 6 years of age (1 = yes)	(−2.13)**	(−0.50)
Children age 6 to 11 years (1 = yes)	−0.150	−0.134
	(−2.84)***	(−2.41)**
Children age 12 to 18 years (1 = yes)	−0.044	−0.042
	(−1.01)	(−0.89)
Children over 18 and away years (1 = yes)	0.088	0.096
	(2.17)**	(2.24)**
**Farm man educational attainment**	0.153	0.091
High school graduate	(2.21)**	(1.62)*
Vocational/technical school/some college	0.211	0.114
	(2.95)***	(1.87)*
College graduate	0.311	0.069
	(4.11)***	(0.96)
Post graduate	0.455	0.332
*Reference category:* Less than high school	(4.98)***	(4.30)***
**Labor market characteristics**	−2.353	−0.703
Commuting zone unemployment rate (2000)	(−2.10)**	(−0.61)
Commuting zone population growth rate (1990–2000)	0.152	0.226
	(0.85)	(1.15)
**Farm inherited or purchased:**	0.015	−0.064
Through her family	(0.33)	(−1.23)
Through his family	0.016	−0.010
	(0.43)	(−0.25)
**Growing up years for spouse/partner**	−0.059	−0.153
In country, but not on farm	(−1.19)	(−2.34)**
In small town	−0.028	−0.158
	(−0.71)	(−3.11)***
Suburban or urban area	−0.026	−0.092
*Reference category:* Growing up on a farm or ranch	(−1.75)*	(−1.53)
**Farm characteristics**	−0.0001	−0.0002
Land owned (acres)	(−4.57)***	(−8.95)***
Debt-asset ratio	0.106	0.059
	(2.14)**	(1.09)
**ERS farm production regions**	−0.034	0.027
Northern Crescent	(−0.72)	(0.53)
Eastern Uplands and Mississippi Portal	0.013	0.105
	(0.27)	(2.08)**
Southern Seaboard	−0.092	0.041
	(−1.53)	(0.64)
Fruitful Rim	−0.039	0.020
	(−0.56)	(0.28)
Basin and Range	−0.036	0.075
*Reference category:* Heartland, Northern Great Plains, Prairie Gateway	(−0.43)	(0.84)

*** = statistically significant at the 0.01 level; ** = significant at 0.05 level; * = significant at 0.10 level.

^1^t-statistics are shown in parentheses.

### Participation in off-farm work: bivariate probit

The marginal effects of age and levels of education that capture the human capital characteristics of the farm woman and her spouse/partner have the anticipated signs and are statistically significant. Thus, participation in off-farm work is increasing with age, reaches a peak and then declines with additional age. The maximum likelihood of off-farm participation is at 37 years of age for the farm woman and 34 years of age for the farm man. Similar results have been obtained by Howard and Swidinsky [40] and Oluwole [41].

The marginal effects for the education variables for the farm women and vocational and post-graduate variables for the farm man (Table 4) are positive and highly significant, indicating a positive relationship between education and off-farm participation. As compared to the less than high school category, the probability of off-farm work for the farm woman is 31.1% higher for those with a 4-year college degree and 45.5% higher for post graduate schooling (Table 4). For the spouse/partner, compared to the less than high school reference category, the likelihood of off-farm work increases by 11.4% for schooling at the vocational/some college level and by 33.2% at the post-graduate level. Hence, increased emphasis on higher education ensures higher productivity in the labor market and translates into higher off-farm participation rates (and higher income).

The presence of children under 6 years of age reduces the probability of off-farm work for the farm woman and the presence of children in the 6 to 11 age group reduces the probability of off-farm work for both the farm woman and spouse/partner (Table 4) The presence of older children (more than 18 and those who have left home) increases the likelihood of working off-farm. Children in the above 18 age category increase the probability of off-farm labor participation by 8.8 percent for the farm woman and 9.6 percent for the spouse/partner.

Farm characteristics are also statistically significant determinants of off-farm labor participation. The debt-asset ratio marginal effect for the overall sample is positive and significant for the farm woman, indicating that the higher the debt-asset ratio, the higher the probability of working in the off-farm labor market (Table 4)^i^. Another variable representing farm characteristics is amount of farm land owned in acres. Results show that amount of farm land owned reduces the likelihood of participation in off-farm work for both the farm woman and her spouse/partner (Table 4). A new variable ‘place of growing up’ was included as an independent variable to test if growing up on a farm as compared to a suburban/urban area affects off-farm work participation. The base or reference category is growing up on a farm or ranch and dummy variables are used. Therefore, the interpretation is in reference to the base category. The ‘place of growing up’ categories were not statistically significant for farm women except for the category of growing up in an urban or suburban environment; suburban/urban women are less likely to participate in off-farm work as compared to women growing up on farms for the overall sample. However, coefficients were very significant for farm men growing up in the country and small towns, indicating that farm men growing up in the country or in a small town are less likely to participate in off-farm work as compared to men growing up on farm or ranch.

Labor market conditions are captured by the commuting zone unemployment rate for the year 2000 and the commuting zone population growth rate^j^. Estimated marginal effects for commuting zone unemployment rate is significant for farm women in the overall sample (Table 4), with the unemployment growth rate being inversely related to off-farm employment. Low unemployment rates and high population growth rates are indicators of a strong economy, encouraging off-farm work.

The USDA Farm Resource Regions have been used principally as indicators of farming systems. The base category consists of the Heartland, Northern Great Plains, and Prairie Gateway, with dummy variables being used to indicate other regions. Table 4 shows husbands/partners in the Eastern Uplands and Mississippi Portal are more likely to be working off-farm as compared to farm men in the base category. The regions used in the base category are characterized by larger farms, and receive large government farm payments [42]. These farms are often operated by the husband/partner and the farm woman is often participating in off-farm work for a variety of reasons ranging from supporting household or farm expenses, receiving non-pecuniary benefits, to being independent and having an own source of income. Interestingly, there was no influence of farm production region on the likelihood of the farm woman participating in off-farm work.

### Benefit receipt: multinomial logit results

Since there was jointness in household decision-making, factors affecting benefit receipt for a household are determined using both farm women’s and spouses off- farm work hours (divided by part time and full time work) in combination with health insurance using a multinomial logit model. The categories are limited to groupings that have sufficient number of observations for estimation. Hence, the status categories for the dependent variable are:

•No off-farm work (Y = 0)

•Farm woman works part-time with no health insurance and spouse/partner works full-time with health insurance (Y = 1)

•Farm woman works full-time with health insurance and spouse/partner works full-time with health insurance (Y = 2)

•Farm woman works full-time with health insurance and spouse/partner works full-time with no health insurance (Y = 3)

•Farm woman works full-time with no health insurance and spouse/partner works full-time with health insurance (Y = 4)

•Farm woman does not work and spouse/partner works full-time with health insurance (Y = 5)

•Farm woman works part-time with no health insurance and spouse/partner does not work (Y = 6)

•Farm woman works full-time with health insurance and spouse/partner does not work (Y = 7)

Table 3 shows the descriptive statistics of variables conditional to the categories defined above. Standard errors of means are presented in parenthesis. Household characteristics included the presence of children between the age’s six to eleven, children between the ages twelve and eighteen and presence children above eighteen or away from home with the reference category being presence of children below the age of six in the households. Educational characteristics of both the farm women and their spouse were included with education less than high school being the reference category. The variables were constructed similar to those of the bivariate probit model on factors affecting decision to work off the farm. Farm characteristic included the amount of land owned by the farm family as an indicator of wealth. We hypothesize that larger amount of farm owned would affect the participation of farm households in off-farm labor markets for health insurance. Total income, household size and an interaction variable of total income and household size are included as an indirect indicator of the impact of the Affordable Care Act on off-farm work decisions. Households with larger total incomes (above 400% of the poverty levels) and small households might have little impact of the policy on working off the farm for health insurance.

Table 5 shows the marginal effects and t-ratios for the multinomial logit model for the farm households in the overall sample. The estimated marginal effects for the categories indicate the effects of the variables on the likelihood or probability of working in the category shown, relative to the omitted category (non-participation or no off-farm work). The multinomial logit categories were tested and found to satisfy the property of independence of irrelevant alternatives (IIA)^k^.

**Table 5:** **Multinomial logit model marginal effects for work status and health insurance categories, 2001 survey**^**1**^

**Variable**	**No off-farm work (Y = 0)**	**Farm woman works part-time with no health insurance & spouse works full-time with health insurance (Y = 1)**	**Farm woman works full-time with health insurance & spouse works full-time with health insurance (Y = 2)**	**Farm woman works full-time with health insurance & spouse works full-time with no health insurance (Y = 3)**
Intercept	−0.353	0.048	0.272	0.026
	(−2.91)***	(1.51)	(4.51)***	(0.65)
Age (years)	0.019	−0.002	−0.007	−0.002
	(11.94)***	(−3.11)***	(−8.69)***	(−3.72)***
**Presence of**:	0.210	−0.007	−0.094	−0.020
Children under 6 years or age 6 to 11 (1 = yes)	(5.38)***	(−0.72)	(−5.24)***	(−1.86)*
Children age 12 to 18 (1 = yes)	−0.032	0.010	−0.009	0.003
	(−0.94)	(1.31)	(−0.67)	(0.31)
Children over 18 and away (1 = yes)	−0.033	0.014	0.004	0.015
	(−1.08)	(1.74)*	(0.31)	(1.84)*
**Farm woman educational attainment**	−0.105	−0.016	0.008	0.017
High school graduate	(−1.59)	(−1.01)	(0.23)	(0.69)
Vocational/technical school/some college	−0.104	−0.023	0.011	0.012
	(−1.51)	(−1.34)	(0.33)	(0.46)
College graduate	−0.171	−0.014	0.038	0.039
	(−2.34)**	(−0.77)	(1.06)	(1.49)
Post graduate	−0.230	0.003	0.082	0.050
*Reference Category: Less than high school*	(−2.83)***	(0.18)	(2.19)**	(1.86)*
**Farm man educational attainment**	−0.119	0.007	0.024	0.018
High school graduate	(−2.54)**	(0.48)	(0.91)	(1.02)
Vocational/technical school/some college	−0.118	0.012	0.038	0.018
	(−2.30)**	(0.81)	(1.37)	(0.94)
College graduate	−0.132	0.021	0.023	0.008
	(−2.28)**	(1.27)	(0.76)	(0.39)
Post graduate	−0.096	−0.003	0.033	0.012
*Reference category: Less than high school*	(−1.42)	(−0.14)	(0.98)	(0.52)
**Farm characteristics**	0.0002	−0.0001	−0.0001	−0.00004
Land owned (acres)	(7.07)***	(−4.17)***	(−3.19)***	(−3.23)***
**Labor market characteristics**	−0.093	−0.013	0.126	0.036
Commuting zone population growth rate (1990–2000)	(−0.68)	(−0.33)	(1.95)**	(0.88)
Commuting zone unemployment rate (2000)	0.541	−0.127	0.105	−0.103
	(0.63)	(−0.50)	(0.24)	(−0.37)
**ERS farm production regions**	0.017	0.002	−0.018	−0.020
Northern Crescent	(0.44)	(0.20)	(−0.96)	(−1.70)*
Eastern Uplands and Mississippi Portal	0.013	−0.006	0.009	−0.007
	(0.36)	(−0.53)	(0.54)	(−0.66)
Southern Seaboard	0.040	−0.014	0.009	−0.013
	(0.82)	(−0.95)	(0.43)	(−0.95)
Fruitful Rim	0.106	0.007	−0.053	−0.018
	(1.89)*	(0.46)	(−1.85)*	(−1.09)
Basin and Range	0.104	−0.008	−0.068	−0.036
	(1.52)	(−0.37)	(−1.74)*	(−1.38)
*Reference category: Heartland, Northern Great Plains, Prairie Gateway*				
**Variable**	**Farm woman works full-time with no health insurance & spouse works full-time with health insurance (Y = 4)**	**Farm woman does not work & spouse works full-time with health insurance (Y = 5)**	**Farm woman works part-time with no health insurance & spouse does not work (Y = 6)**	**Farm woman works full-time with health insurance & spouse does not work (Y = 7)**
Intercept	0.027	0.066	−0.081	−0.005
	(0.66)	(1.14)	(−1.72)*	(−0.06)
Age (years)	−0.002	−0.003	0.001	−0.004
	(−3.78)***	(−4.03)***	(1.09)	(−4.23)***
**Presence of**:	−0.017	−0.014	0.004	−0.062
Children under 6 years or age 6 to 11 (1 = yes)	(−1.68)*	(−0.73)	(0.25)	(−2.84)***
Children age 12 to 18 (1 = yes)	0.005	−0.009	0.020	0.011
	(0.66)	(−0.57)	(1.39)	(0.64)
Children over 18 and away (1 = yes)	0.017	−0.009	−0.016	0.007
	(2.12)**	(−0.60)	(−1.13)	(0.42)
**Farm woman educational attainment**	0.002	−0.024	−0.012	0.130
High school graduate	(0.10)	(−0.82)	(−0.54)	(2.14)**
Vocational/technical school/some college	−0.007	−0.029	−0.010	0.149
	(−0.32)	(−0.96)	(−0.42)	(2.44)**
College graduate	−0.004	−0.043	−0.002	0.158
	(−0.18)	(−1.30)	(−0.08)	(2.53)**
Post graduate	0.013	−0.122	−0.040	0.245
*Reference category: Less than high school*	(0.54)	(−2.83)***	(−1.08)	(3.97)***
**Farm man educational attainment**	0.056	0.027	−0.015	0.003
High school graduate	(2.41)**	(1.03)	(−0.87)	(0.09)
Vocational/technical school/some college	0.055	0.038	−0.003	−0.040
	(2.28)**	(1.35)	(−0.15)	(−1.31)
College graduate	0.059	0.056	−0.013	−0.022
	(2.34)**	(1.81)*	(−0.58)	(−0.66)
Post graduate	0.047	0.131	−0.076	−0.048
*Reference Category: Less than high school*	(1.68)*	(4.14)***	(−1.98)**	(−1.22)
**Farm characteristics**	−0.00005	−0.00004	0.00002	0.00003
Land owned (acres)	(−3.46)***	(−1.82)*	(4.95)***	(4.17)***
**Labor market characteristics**	−0.063	−0.037	−0.027	0.072
Commuting zone population growth rate (1990–2000)	(−1.43)	(−0.52)	(−0.46)	(0.95)
Commuting zone unemployment rate (2000)	−0.431	0.324	−0.288	−0.021
	(−1.49)	(0.76)	(−0.77)	(−0.04)
**ERS farm production regions**	0.002	0.031	0.006	−0.020
Northern Crescent	(0.18)	(1.62)*	(0.39)	(−0.96)
Eastern Uplands and Mississippi Portal	0.011	0.037	−0.014	−0.044
	(1.00)	(1.95)**	(−0.78)	(−1.98)**
Southern Seaboard	−0.024	0.059	−0.038	−0.018
	(−1.28)	(2.59)***	(−1.42)	(−0.67)
Fruitful Rim	0.004	0.010	0.014	−0.068
	(0.23)	(0.32)	(0.59)	(−1.96)*
Basin and Range	0.008	0.032	0.026	−0.058
	(0.38)	(0.97)	(1.00)	(−1.39)
*Reference category: Heartland, Northern Great Plains, Prairie Gateway*				
**Model Performance Indicators**				
Number of observations : 2176 Log likelihood function: -3225.244 Restricted log likelihood: -3446.011				
Chi squared : 441.5335***				

*** = statistically significant at the 0.01 level; ** = significant at 0.05 level; * = significant at 0.10 level.

^1^t-statistics are shown in parentheses.

Among the individual characteristics, age is found to be statistically significant for all the work choice categories implying that the likelihood of participation in off-farm decreases with increasing age (Table 5). Age is not found to influence work choice category where farm woman works part-time with no health insurance and spouse/partner works on the farm (Y = 6), but education strongly affects the off-farm jobs taken by those who farm. Households in which both farm woman and spouse/partner are working off-farm (Y = 2 and Y = 3), farm women with post graduate degrees are more likely to be working at full-time jobs with health insurance (Table 5). However, in households where only the farm woman is working at an off-farm job and spouse/partner is not working (Y = 7), farm women with high school, vocational, college or post graduate degrees are more likely to be working at full-time at jobs with health insurance, making sure that at least one spouse brings in the non-pecuniary benefits for the farm household (Table 5). Similarly, households in which the spouse/partner is working full-time with health insurance, are more likely to be the ones (Y = 4 and Y = 5), with college degrees and post graduate degrees, as opposed to the ones who are working full-time without benefits or not working at all (Table 5). These results are consistent with the findings of the past studies, in which level of education increases the likelihood of finding jobs with benefits.

Farm households work decisions are interrelated across the household and they adjust their labor participation according to demands of the situation. This is clearly evident when we look at household characteristics represented by the presence of children. The results show that if children are present in the age-group less than 6 years old or 6-11years, it is more likely for the farm woman to stay at home or on the farm (Y = 0) and less likely to have an off-farm job (Y = 2, Y = 3, Y = 4, and Y = 7) while the husband is working at a full time job with or without health insurance or not working at all. However, once the children are older, farm families make sure that there is health insurance for the family, either through the farm woman’s job or through the spouse’s off-farm job, with the other parent adjusting according to the needs of the family.

The statistically significant coefficients for the category ‘children above 18 and away’ implies that farm women are more likely to be working part-time with no health insurance or full-time with or without health insurance, *as long as the husband is working with health insurance* (Y = 1,Y = 3,Y = 4). It is interesting to note that farm women are always more likely to be working (part-time or full-time) in the labor market, even if the husband receives benefits, and definitely full-time with health insurance if spouse/partner not receiving benefits, when the children are older. This might be because of increased expenditures and since children are now school-age, farm women are working off-farm to support household expenditures or since there are older children in family, there might be labor substitutability and farm women can work off-farm.

Evidence from past studies has shown that farm size (farm asset value) is inversely proportional to participation in off-farm work. Results from this study also re-enforce similar conclusions. Hence, if the amount of land owned is large, off-farm work is less likely as can be seen from statistically significant marginal coefficients when Y = 1, Y = 2, Y = 3, Y = 4, or Y = 5. However, despite operating large farm, farm women may still work at part-time jobs, perhaps for social or professional reasons like maintaining their skills [26]. This sentiment is definitely supported by our results for households (Y = 6) in which farm women are working part-time with no benefits and the spouse has no off-farm job. However, if the spouse has no off-farm job (Y = 7) then the farm women is most likely to be working at full-time job that provides health insurance, thereby saving money from buying benefits for the huge farm operation as indicated by positive and significant coefficient of land owned in acres. Hence, while the spouse/partner takes care of the farm, the farm women makes sure that the employer-provided non-pecuniary benefits might be helping to finance the household expenses (in terms of material comforts of life) or farm expenses and non-pecuniary benefits help avoid unforeseen health care expenditures. The above conclusion is yet another example of joint decision making in farm households and dynamic adjustment depending on the needs of the situation.

According to a study by Meyer *et al.*, [38] a strong economy implies a strong local labor market that leads to increases in labor demand, whereas high unemployment rates and a low population growth rate may cause the firms to reduce benefit packages with high fixed costs. Two major interrelated results from this study point towards a robust local labor market. One is the direct result that a statistically positive commuting zone population growth rate increase the likelihood that both the farm woman and spouse/partner work at full-time jobs with health benefits (Y = 2) (Table 5). The other, a more indirect result, is the likely presence of high-wage firms in these markets. These firms tie benefits to earnings of workers and avoid hiring low-wage workers, as it has to offer all full-time workers same benefits (IRS ruling). The fact that both farm women and spouse/partner have full-time jobs with benefits is an indicator of high average earnings, and hence a robust local economy.

In terms of regional variation, with reference to the base category which consists of the Heartland, Northern Great Plains, Prairie Gateway, households where the husband/partner works full-time with health insurance and the farm woman does not work in the off-farm labor market (Y = 5) have a high probability of being located in the Northern Crescent, Eastern Uplands, Mississippi Portal and Southern Seaboard. The result is consistent with the findings in a report released by Eathington and Swenson [42]. Most farms in the Eastern Uplands and Southern Seaboard are family farms associated with poultry production and part-time cattle. On dairy farms (Northern Crescent), work remains intense throughout the season and women are found to be working more than 40 hours per week on the farm (Farm Women Survey, 2001^l^). The Eastern Uplands region has the lowest weighted average sales value (Census of Agriculture, 1997). As a result, most farm operators depend on off-farm employment to supplement their incomes and also receive benefits. Hence, farm women tend to take care of the farms while the spouse/partner works at off-farm jobs with benefits. The Fruitful Rim region has the highest weighted average value of sales per farm, followed by farms in the heartland which has the highest volume of sales. As a result, the Fruitful Rim and Heartland have largest numbers of farm jobs and also receive the highest overall average government payments per farm [42]. The ‘Women on Farm Survey’ (2001) also shows that these (Northern Crescent, Fruitful Rim, Northern Great Plains) are the regions which emphasize crops and where women are working 40 hours/week in the spring, summer and fall. Hence, households where both spouses are working full-time off-farm with health insurance (Y = 2) or where wife works full-time with health insurance and the husband does not work (Y = 7) are less likely to be located in the Fruitful Rim and Eastern Uplands regions.

To explore the impact of the new health care policy on farm household’s decision making process of working off the farm; we attempted to use three indirect measures of eligibility to participate in health exchanges. Total Income, household size and an interaction of the two variables. We expected those households with incomes between 133% and 150% of the poverty line and who either do not work off the farm or work off the farm with no health insurance benefits would show a negative correlation to working off the farm in light of the availability of health insurance exchanges. Results were inconclusive since the hessian matrix in the maximum likelihood estimation was singular and we had to drop these variables. Since the survey was developed earlier to such policy discussions and the enacted law, the design of the survey was limited for further analysis. Additionally, the law might affect differently in different states based on development of state health exchanges or participation in national exchanges. While certain assumptions on the behavior of farm families can be made based on the survey an *ex-post* analysis on the impact of the Affordable Care Act on farm decision making is out of scope of this paper.

## Conclusion

Receiving benefits like health insurance or life insurance with an off-farm job is a very important reason to participate in off-farm work, both for farm women and farm men. It is especially important for farm women if the husband works on the farm. But there are very few studies on farm households and impact of health insurance on labor outcomes. This paper tries to understand factors affecting choice of work (taking into account the joint decisions of farm women and farm men) when both pecuniary and non-pecuniary returns to work are considered in conjunction with hours of off-farm work. First, a bivariate probit model is estimated to test jointness in decisions related to off-farm work participation and because off-farm work decisions by the farm woman and spouse/partner are shown to be correlated, an integrated model using multinomial logit is estimated to analyze work choices taking health benefits into account The method of maximum likelihood is used to estimate the coefficients and marginal effects of the regressors.

The study shows the impacts of individual characteristics (age and age square), human capital (levels of education), farm (land owned) and family characteristics (children in different age groups), and labor market (commuting zone population growth rate and commuting zone unemployment rate) variables on the joint work decisions of the farm woman and her spouse/partner in adjusting their off-farm work statuses-- working full-time or part-time (in combination or individually)-- to receive health insurance from off-farm employment. The results of this study re-enforce findings from other studies [24] that if the spouse/partner is working full-time with health insurance, then the farm women is more likely to work part-time or full-time without insurance perhaps to socialize with other people or be independent, depending on the situation of the farm household. Hence, to avoid the (high) expense of purchasing health insurance, farm families seek off-farm jobs with health insurance.

Farm households continue to diversify their economic activities; hence, it is important to understand the motivations, means and outcomes of this heterogeneous diversifying process. The results of the study have important implications for policy makers to understand joint decision making in a farm household and factors affecting benefit receipt to come up with affordable alternatives to employer provided health insurance plans or appropriate rural development initiatives to facilitate more off-farm job opportunities. In fact, current versions of the health care reform bills which extend coverage to un-insured people might increase access to various health care plans, both in terms of choice and cost to farm households in US.

## Endnotes

^a^For example, if a firm wishes to offer an executive a defined benefit pension plan that defers compensation, the firm must offer her secretary a similar plan.

^b^The study by Olson [24] uses March 1993 Current Population Survey data and uses parametric and semi-parametric statistical models to show that wives without spousal health benefits are more likely to work full-time than those who do have spousal benefits.

^c^The study by Buchmuellar and Valetta [25] uses the April 1993 Current Population Survey Benefits Supplement to investigate the effects of employer-provided health insurance on labor supply of married women.

^d^Questions asked in the survey about the farm woman and her spouse/partner were:

Question 30: Did you work in any non-farm wage or salary job at all during 2000?

▪ Yes

▪ No

Question 33: Did your husband/partner work at any non-farm wage or salary job at all during 2000?

▪ Yes

▪ No

^e^Husband/partner are synonymous with spouse/partner.

^f^Part-time is defined as working less than 35 hours in an off-farm job. Full-time is defined as working between 35 and 40 hours, inclusive.

^g^Q48A Including yourself, how many people live in your household?

Q48 What was your total household income before taxes and other withholdings in 2000 (including farm income, off-farm income, dividends, rent, and any other income)?

1) Less than $10,000 2) $10,000-$19,999 3) $20,000- $29,999 4) $30,000- $39,999 5) $40,000- $49,999

6) $50,000- $74,999 7) $75,000- $99,999 8) $100,000- $249,999 9) $250,000- $499,999 10) $500,000 and above

^h^Very often it is found that individuals travel to neighboring counties for work. Commuting zones are constructed using, journey-to-work data and define clusters of counties with strong commuting ties. The statistical method of cluster analysis is used to assign each county to a single commuting zone based on the journey-to-work commuting data. The journey-to-work data identify the county workplace destination for residents of all U.S. counties and equivalents. (See C. Tolbert and M. Sizer, U.S. Commuting Zones and Labor Market Areas, for additional information on the statistical methods used to construct commuting zones). The present dataset was merged with commuting zones to arrive at commuting zone unemployment and population growth rates.

^i^The survey asked respondents ranges of the debt and ranges of value of assets the farm households owned. For the participation model we used the mean of each of the ranges asked to create a debt asset ratio variable. However introducing the variable for the multinomial logit regression caused the hessian matrix to be singular, this might be due to the fact that the distribution of the responses was very low or not available for some of the categories defined.

^j^Growth rate is calculated over a ten year period from 1990 to 2000.

^k^The IIA assumption was tested using the Hausman test. Support was found for the null hypothesis that the categories are independent of each other.

^l^Information about hours spent in a week working on farms in spring, summer and fall were accessed at from the following website: http://ageconsearch.umn.edu/bitstream/33470/1/fo02fi01.pdf.

## Competing interests

Authors declare that they have no competing interests.

## Authors’ contributions

LB contributed to conceptualizing the research idea and study design, literature search, data analysis, data interpretation, writing, revising and finalizing the manuscript. JF contributed to data analysis, data interpretation, writing the manuscript. SC contributed to data analysis and, writing the manuscript. All the authors read and approved the final manuscript.
